# MEIS2 is essential for neuroblastoma cell survival and proliferation by transcriptional control of M-phase progression

**DOI:** 10.1038/cddis.2014.370

**Published:** 2014-09-11

**Authors:** Y Zha, Y Xia, J Ding, J-H Choi, L Yang, Z Dong, C Yan, S Huang, H-F Ding

**Affiliations:** 1 Department of Neurology, The First Hospital of Yichang, Institute of Translational Neuroscience, Three Gorges University College of Medicine, Yichang, China; 2 Cancer Center, Georgia Regents University, Augusta, GA, USA; 3 Department of Biostatistics and Epidemiology, Georgia Regents University, Augusta, GA, USA; 4 State Key Laboratory of Silkworm Genome Biology, Institute of Sericulture and System Biology, Southwest University, Chongqing, China; 5 Department of Cellular Biology and Anatomy, Georgia Regents University, Augusta, GA, USA; 6 Department of Biochemistry and Molecular Biology, Georgia Regents University, Augusta, GA, USA; 7 Department of Pathology, Medical College of Georgia, Georgia Regents University, Augusta, GA, USA

## Abstract

MEIS2 has an important role in development and organogenesis, and is implicated in the pathogenesis of human cancer. The molecular basis of MEIS2 action in tumorigenesis is not clear. Here, we show that MEIS2 is highly expressed in human neuroblastoma cell lines and is required for neuroblastoma cell survival and proliferation. Depletion of MEIS2 in neuroblastoma cells leads to M-phase arrest and mitotic catastrophe, whereas ectopic expression of MEIS2 markedly enhances neuroblastoma cell proliferation, anchorage-independent growth, and tumorigenicity. Gene expression profiling reveals an essential role of MEIS2 in maintaining the expression of a large number of late cell-cycle genes, including those required for DNA replication, G2-M checkpoint control and M-phase progression. Importantly, we identify MEIS2 as a transcription activator of the MuvB-BMYB-FOXM1 complex that functions as a master regulator of cell-cycle gene expression. Further, we show that FOXM1 is a direct target gene of MEIS2 and is required for MEIS2 to upregulate mitotic genes. These findings link a developmentally important gene to the control of cell proliferation and suggest that high MEIS2 expression is a molecular mechanism for high expression of mitotic genes that is frequently observed in cancers of poor prognosis.

Myeloid ecotropic insertion site 2 (MEIS2) is a member of the three amino-acid loop extension (TALE) family of homeodomain-containing transcription factors that function as regulators of cell proliferation and differentiation during development, and are involved in proximal-distal limb patterning, skeletal muscle differentiation, and the development of hindbrain, lens, and retina.^1, 2, 3, 4, 5, 6, 7, 8^ All mammalian MEIS proteins (MEIS1–3) contain a conserved Hth (homothorax) domain originally identified in the *Drosophila* Hth protein, which mediates the interaction with other homeodomain proteins and binds to DNA sequences containing a conserved TGACAG motif. MEIS2 also has a C-terminal transcriptional activation domain, which is required for full activation of transcription by homeodomain protein complexes containing MEIS2.^9^

Accumulated evidence suggests an oncogenic role for MEIS proteins in the development of human cancers. The *MEIS1* gene is amplified and overexpressed in ovarian cancers compared with normal ovarian surface epithelium.^10, 11^ In lung cancer, MEIS1/2-mediated downregulation of TGF-*β* type II receptor expression has been suggested as a major mechanism for inactivation of transforming growth factor *β* (TGF-*β*)-induced tumor suppressor function.^12^ In leukemia, it has been shown that MEIS1 cooperates with HOXA9 in transforming mouse bone marrow cells, leading to acute myeloid leukemia.^13^ In addition, MEIS1/2 expression is essential for maintaining myeloid cell lines in an undifferentiated, proliferating state by inhibiting myeloid differentiation.^14, 15^

Amplification or high-level expression of MEIS1 and MEIS2 has been also detected in primary tumors and cell lines of neuroblastoma,^16, 17^ a common childhood malignant tumor of the sympathetic nervous system.^18, 19^ However, the functional significance of MEIS genes in neuroblastoma is not clear. In this study, we investigated the function of MEIS2 in neuroblastoma cells and the underlying molecular mechanism. Our investigation uncovered a critical role of MEIS2 in the control of late cell-cycle genes through transcriptional activation of FOXM1 and its associated proteins that function as a master regulator of cell-cycle gene expression.^20, 21^

## Results

### MEIS2 is essential for the survival of neuroblastoma cells

**Figure 1:**
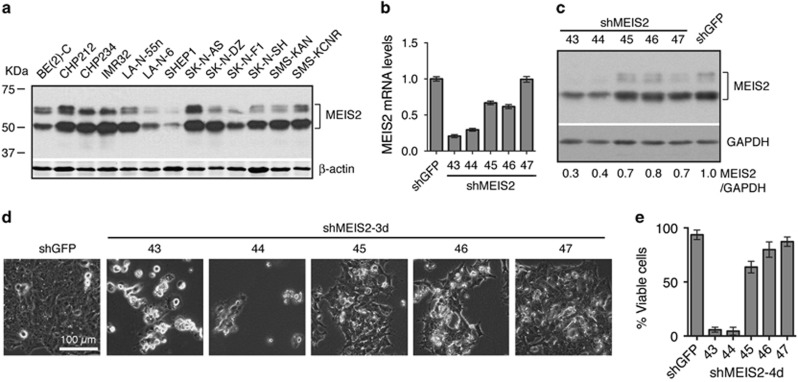
MEIS2 is essential for neuroblastoma cell survival. (**a**) Immunoblotting of MEIS2 in a panel of 13 human neuroblastoma cell lines. *β*-Actin levels are shown as a loading control. (**b** and **c**) qRT-PCR (**b**) and immunoblot (**c**) analyses of MEIS2 expression in BE(2)-C cells infected with lentiviruses expressing either shGFP or shMEIS2. Error bars (**b**) represent S.D. (*n*=3). GAPDH (**c**) serves as a loading control. (**d**) Micrographs of BE(2)-C cells infected for 3 days with lentiviruses expressing either shGFP or shMEIS2. (**e**) Trypan blue exclusion assay of viable BE(2)-C cells infected for 4 days with lentiviruses expressing either shGFP or shMEIS2

Human cells express 7 MEIS2 protein isoforms (a–d and f–h) composed of 306–477 amino acids and one non-coding RNA product, MEIS2e (Supplementary Figure S1). Consistent with previous reports,^16, 17^ immunoblot analysis of a panel of 13 human neuroblastoma cell lines revealed that they all expressed significant levels of MEIS2 isoforms with apparent molecular weight of 46–60 kDa (Figure 1a). To examine the functional significance of high-level MEIS2 expression, we silenced MEIS2 expression in the neuroblastoma cell line BE(2)-C cells using small-hairpin RNA (shRNA). Among the five shMEIS2 constructs tested, two (shMEIS2-43 and-44) were highly effective in knockdown of MEIS2 expression (>60%), as determined by quantitative RT-PCR (qRT-PCR) (Figure 1b) and immunoblotting (Figure 1c). MEIS2 depletion in BE(2)-C cells resulted in a marked reduction in cell survival, with > 90% of the cells losing viability within 4 days of infection with lentiviruses expressing either shMEIS2-43 or shMEIS2-44 (Figures 1d and e). We obtained essentially the same results with three additional neuroblastoma cell lines, SK-N-DZ, SK-N-FI, and SHEP1 (Supplementary Figure S2). Collectively, these findings indicate that high-level expression of MEIS2 is essential for the survival of neuroblastoma cells.

### MEIS2 depletion induces mitotic catastrophe

We next investigated the nature of cell death induced by MEIS2 depletion. We performed several assays to determine whether these cells died by apoptosis. BE(2)-C cells infected with lentiviruses expressing either shMEIS2-43 or shMEIS2-44 showed no sign of genomic DNA fragmentation or cleavage of pro-caspase 3 (Supplementary Figures S3a and b), two hallmarks of apoptosis.^22^ We also generated BE(2)-C cells overexpressing BCL-2 (Supplementary Figure S3c), a well-characterized anti-apoptotic protein.^23^ BCL-2 overexpression failed to protect BE(2)-C cells from shMEIS2-43-induced cell death (Supplementary Figure S3d). Similarly, treatment of BE(2)-C cells expressing either shMEIS2-43 or shMEIS2-44 with the cell permeable pan-caspase inhibitor zVAD-FMK failed to prevent cell death although it was highly effective in protecting cells from Fas-mediated apoptosis (Supplementary Figure S3e). Together, these data indicate that MEIS2 depletion induces predominantly non-apoptotic cell death in neuroblastoma cells.

**Figure 2:**
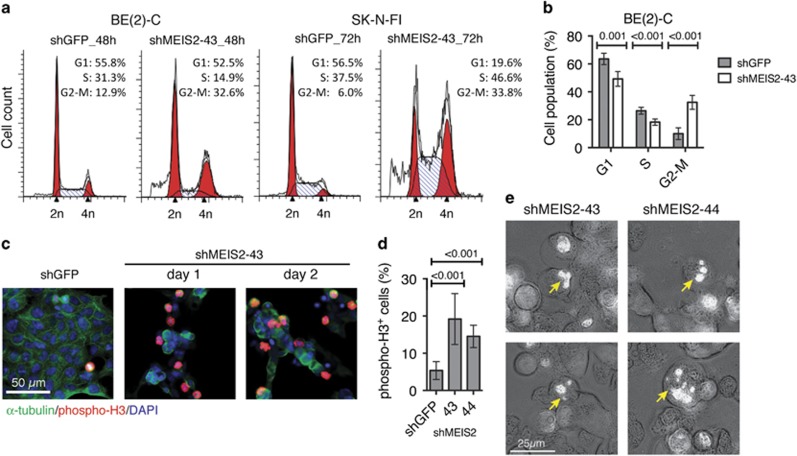
MEIS2 depletion induces M-phase arrest and mitotic catastrophe. (**a** and **b**) Cell-cycle analysis (**a**) and quantification (**b**) showing that MEIS2 depletion induces M-phase arrest in BE(2)-C and SK-N-F1 cells. Error bars (**b**) represent S.D. (*n*=3). (**c** and **d**) Immunofluorescence staining (**c**) and quantification (**d**) of phospho-histone H3^+^ M-phase BE(2)-C cells following infection with lentiviruses expressing either shGFP or shMEIS2-43. Error bars (**d**) represent S.D. Approximately 400–1000 cells (DAPI positive) were counted from at least 10 random × 630 fields. (**e**) Hoechst 33342 staining of nuclei showing chromatin condensation and micronucleation (arrows) in BE(2)-C cells following infection with lentiviruses expressing shMEIS2-43 or shMEIS2-44. Data (**b** and **d**) were analyzed with two-tailed Student's *t*-test with the *P*-values indicated

We noted that following the infection with lentiviruses expressing either shMEIS2-43 or shMEIS2-44, there was a dramatic increase in the number of cells that appeared round and refractile (Supplementary Figure S4a), which morphologically resembled the cells treated with Nocodazole (Supplementary Figure S4b), an anti-cancer agent that induces M-phase arrest by interfering with the polymerization of microtubules. We confirmed that MEIS2 depletion in both BE(2)-C and SK-N-FI cells induced M-phase arrest by cell-cycle analysis (Figures 2a and b) and by immunofluorescence staining for phosphorylated histone H3 at Ser10 (phospho-H3, Figures 2c and d), a marker for mitotic cells.^24^ In addition, Hoechst 33342 staining of nuclei revealed chromatin condensation and micronucleation (Figure 2e; Supplementary Figure S4c). Micronucleation results from asymmetric distribution of chromosomes or chromosome fragmentation and is a morphological feature of mitotic catastrophe.^25, 26^ Thus, MEIS2 depletion in neuroblastoma cells triggers non-apoptotic cell death with morphologic features of mitotic catastrophe.

### MEIS2 depletion induces mitotic spindle aberrations and centrosome amplification

**Figure 3:**
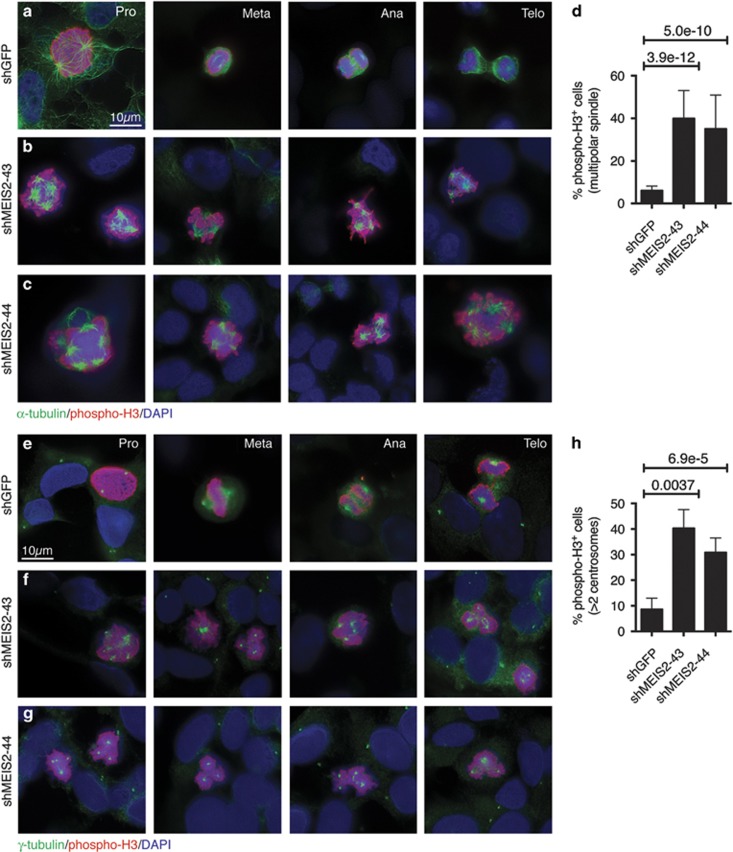
MEIS2 depletion induces multipolar mitotic spindles and centrosome amplification. (**a**–**c**) Immunofluorescence staining for M-phase BE(2)-C cells (phospho-H3^+^) and mitotic spindles (*α*-tubulin) following infection with lentiviruses expressing shGFP (**a**), shMEIS2-43 (**b**), or shMEIS2-44 (**c**). (**d**) Quantification of M-phase BE(2)-C cells (phospho-H3^+^) with multipolar spindles. (**e**–**g**) Immunofluorescence staining for M-phase BE(2)-C cells (phospho-H3^+^) and centrosomes (*γ*-tubulin) following infection with lentiviruses expressing shGFP (**e**), shMEIS2-43 (**f**), or shMEIS2-44 (**g**). (**h**) Quantification of M-phase BE(2)-C cells (phospho-H3^+^) with more than two centrosomes. Error bars (**d** and **h**) represent S.D. Approximately 400–1000 cells (DAPI positive) were counted from at least 10 random × 630 fields. Data (**d** and **h**) were analyzed with two-tailed Student's *t*-test with the *P*-values indicated. Pro, prophase; Meta, metaphase; Ana, anaphase; and Telo, telophase

Mitotic catastrophe is generally thought to result from aberrant mitosis such as disruption of the mitotic spindle function or structure that ensures the correct segregation of chromosomes between daughter cells.^26, 27^ Therefore, we further characterized these mitotic cells by co-immunofluorescence staining with antibodies against phospho-H3 and *α*-tubulin, a component of mitotic spindles.^28^ A majority of control mitotic cells displayed a typical bipolar spindle and they were found at different stages of mitosis: prophase, metaphase, anaphase, and telophase (Figure 3a). By contrast, very few mitotic cells with MEIS2 depletion underwent cytokinesis and ∼40% of them displayed multiple spindle poles (Figures 3b–d). Given the critical role of the centrosome in mitotic spindle organization,^29^ we also examined the structure of centrosomes in the mitotic cells by co-immunofluorescence staining for phospho-H3 and *γ*-tubulin, a component of the centrosome.^30^ More than 90% of control phospho-H3^+^ cells contained two centrosomes (Figures 3e and h), whereas between 30 and 40% of phospho-H3^+^ cells with MEIS2 depletion displayed more than two centrosomes (Figures 3f–h). Thus, MEIS2 depletion triggered centrosome amplification, leading to the formation of multipolar spindles and, consequently, mitotic catastrophe.

### MEIS2 depletion leads to global downregulation of late cell-cycle genes

To gain a molecular understanding of MEIS2 depletion-induced mitotic catastrophe, we conducted microarray gene expression profiling of BE(2)-C cells at 48 h after infection with lentiviruses expressing either shGFP or shMEIS2-43. The profiling analysis identified a total of 1206 genes that were differentially expressed (≥ +1.5 and ≤−1.5 fold, *P*<0.01), with 546 genes being upregulated and 660 genes downregulated (Supplementary Table S2). Gene ontology (GO) analysis of the 546 genes upregulated by shMEIS-43 revealed no clear explanation for the phenotype of M-phase arrest and mitotic catastrophe induced by MEIS2 depletion (Supplementary Table S3).

**Figure 4:**
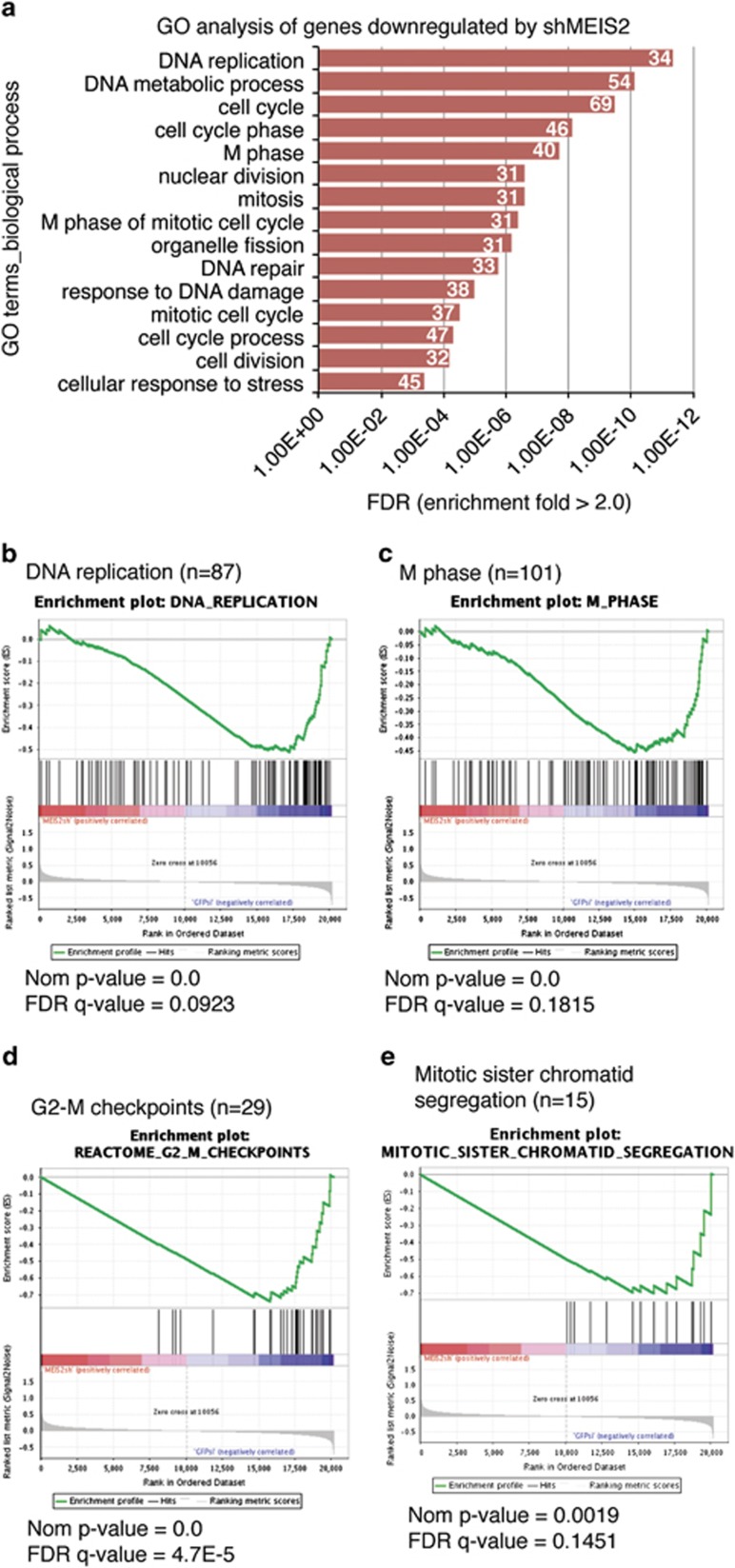
MEIS2 depletion leads to global downregulation of late cell-cycle genes. (**a**) GO analysis of the genes downregulated by shMEIS2-43 in BE(2)-C cells (FDR<1%). The number of genes for each biological process category is indicated. (**b**–**e**) GSEA showing significant enrichment of the gene sets involved in DNA replication (**b**), M phase (**c**), G2-M checkpoints (**d**), and mitotic sister chromatid segregation (**e**), with a majority of these genes being downregulated in response to MEIS2 depletion

By contrast, GO analysis of the 660 downregulated genes revealed that they were remarkably enriched for genes that control the cell cycle (Figure 4a; Supplementary Table S4, enrichment fold≥2.0; FDR ≤1%), with 34 genes being involved in DNA replication and 40 genes in the M phase (Figure 4a). Similarly, GSEA showed that among the genes downregulated by shMEIS2-43, those regulating DNA replication (Figure 4b) and mitosis (Figure 4c) were significantly enriched. Particularly interesting is the downregulation of genes that control the G2-M checkpoint (Figure 4d; Supplementary Figure S5a) and mitotic sister chromatid segregation (Figure 4e; Supplementary Figure S5b). It is known that defects in the G2-M checkpoint control and mitotic sister chromatid segregation are major causes of mitotic catastrophe.^31, 32^ These gene expression profiling data suggest that MEIS2 depletion triggers aberrant mitosis and mitotic catastrophe by transcriptional repression of a large number of genes critical for DNA replication, G2-M checkpoint control, and M-phase progression. Thus, MEIS2 at its steady-state levels has an essential role in the transcriptional activation of key cell-cycle genes, at least in neuroblastoma cells.

### MEIS2 targets the MuvB-BMYB-FOXM1 complex for transcriptional control of M-phase progression

**Figure 5:**
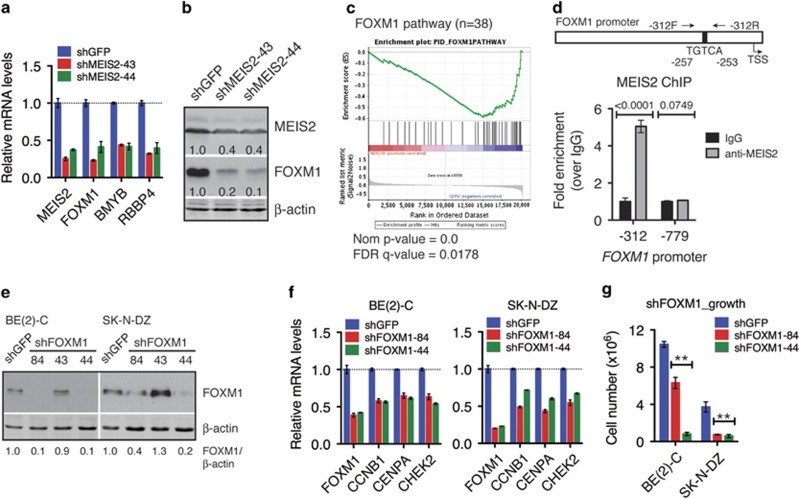
MEIS2 targets the MuvB-BMYB-FOXM1 complex for transcriptional control of M-phase progression. (**a**) qRT-PCR analysis of mRNA expression of MEIS2, FOXM1, BMYB, and RBBP4 in BE(2)-C cells infected with lentiviruses expressing either shGFP or shMEIS2. (**b**) Immunoblotting of MEIS2 and FOXM1 in BE(2)-C cells infected with lentiviruses expressing either shGFP or shMEIS2. *β*-Actin levels are shown as a loading control. MEIS2 and FOXM1 levels were quantified against *β*-actin. (**c**) GSEA showing marked downregulation of the FOXM1 pathway genes in BE(2)-C cells with MEIS2 depletion. (**d**) ChIP-qPCR analysis showing MEIS2 binding to the *FOXM1* promoter region (−312 to −158) containing a consensus TGIF/MEIS2-binding sequence TGTCA. Error bars, S.D. (*n*=3). (**e**) Immunoblotting of FOXM1 in BE(2)-C and SK-N-DZ cells with or without FOXM1 knockdown. *β*-Actin levels are shown as a loading control. (**f**) qRT-PCR analysis of mRNA expression of key FOXM1 target genes in BE(2)-C and SK-N-DZ cells with or without FOXM1 knockdown. (**g**) Growth assay of BE(2)-C and SK-N-DZ cells with or without FOXM1 knockdown. Error bars, S.D. (*n*=3). **P<0.001. Data (**d** and **g**) were analyzed with two-tailed Student's *t*-test

It has been reported recently that the MuvB (multi-vulval class B) complex, which is composed of LIN9, LIN37, LIN52, LIN54, and RBBP4, has an essential role in coordinating the expression of cell-cycle genes for orderly cell-cycle progression.^21^ The MuvB complex recruits BMYB, also known as MYBL2, to the promoters of S-phase cell-cycle genes to activate their transcription, and the MuvB-BMYB complex subsequently recruits Forkhead box protein M1 (FOXM1) to the promoters of G2-M phase cell-cycle genes for their activation.^21, 33, 34^ Our microarray profiling revealed that the expression of RBBP4, BMYB, and FOXM1 was significantly downregulated following MEIS2 depletion (Supplementary Tables S2 and S4). We confirmed the result by qRT-PCR (Figure 5a). Also, immunoblot analysis revealed a significant decrease in FOXM1 protein levels following MEIS2 knockdown (Figure 5b). Moreover, canonic pathway analysis of shMEIS2-responisve genes by GESA revealed that the FOXM1 pathway gene set was significantly enriched (Figure 5c). Among the 38 pathway genes, the expression levels of 25 genes, including FOXM1, were significantly reduced following MEIS2 depletion as determined by microarray profiling (Supplementary Figure S6a). The downregulation of FOXM1 target genes was also confirmed by qRT-PCR (Supplementary Figure 6b). It is known that cells with FOXM1 depletion display mitotic spindle defects, centrosome amplification, chromosome mis-segregation, failure of cytokinesis, and mitotic catastrophe,^35, 36, 37^ which are essentially the same phenotypes displayed by neuroblastoma cells with MEIS2 depletion as described above. In addition, it has been reported recently that FOXM1 is overexpressed in primary neuroblastoma tumors and is essential for the tumorigenicity of neuroblastoma cells.^38^ Consistent with the report, we found that high FOXM1 expression is significantly associated with poor prognosis and advanced tumor stages in neuroblastoma patients (Supplementary Figures S6c and d).

Chromatin immunoprecipitation (ChIP)-qPCR analysis showed specific interaction of endogenous MEIS2 with the *FOXM1* promoter region spanning from −312 to −158 (5.1-fold enrichment relative to IgG control), which contains a consensus TGIF/MEIS2-binding sequence TGTCA^39^ 257 bp upstream of the transcription start site (TSS) (Figure 5d). The same ChIP-qPCR assay revealed no significant association of MEIS2 with the promoter regions of *RBBP4* and *BMYB* (data not shown). These findings, in combination with gene expression data, indicate that *FOXM1* is a direct target gene of MEIS2, whereas the expression of RBBP4 and BMYB appears to be indirectly regulated by MEIS2.

In agreement with previous findings,^35, 36, 37^ shRNA-mediated depletion of FOXM1 in BE(2)-C and SK-N-DZ cells (Figure 5e) resulted in marked downregulation of known FOXM1 target genes (Figure 5f) and loss of cell viability as a result of mitotic catastrophe (Figure 5g; Supplementary Figures S7a and b). Similarly, treatment of BE(2)-C and SK-N-DZ cells with siomycin A or thiostrepton, specific inhibitors of FOXM1,^40, 41, 42^ led to cell death with morphological features of mitotic catastrophe (Supplementary Figure S7c). Thus, FOXM1 depletion or inhibition in neuroblastoma cells fully recapitulated the phenotype of MEIS2 depletion at both molecular and cellular levels. Collectively, these data suggest that MEIS2 depletion-induced mitotic catastrophe results from the downregulation of FOXM1, RBBP4 and BMYB, which in turn leads to the downregulation of mitotic genes and disruption of M-phase progression.

### MEIS2 promotes the proliferation and tumorigenicity of neuroblastoma cells

**Figure 6:**
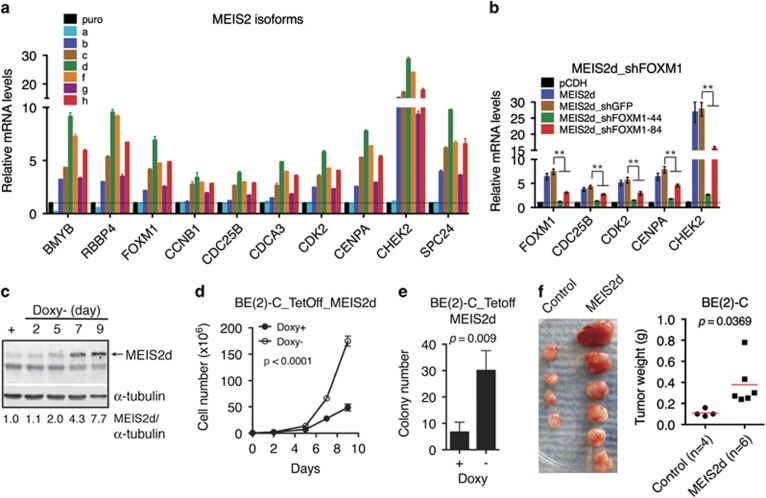
MEIS2 enhances the proliferation and tumorigenicity of neuroblastoma cells. (**a**) qRT-PCR analysis of mRNA expression of RBBP4, BMYB, FOXM1 and their target genes in BE(2)-C cells transfected with plasmids expressing individual MEIS2 isoforms. Error bars, S.D. (*n*=3). (**b**) qRT-PCR analysis of mRNA expression of FOXM1 and its key target genes in BE(2)-C cells co-transfected with an MEIS2d-expessing plasmid along with constructs expressing shGFP, shFOXM1-44, or shFOXM1-84. Error bars, S.D. (*n*=3). **P<0.01. (**c**) Immunoblotting of MEIS2d in BE(2)-C_tetoff_MEIS2d cells with or without doxycycline (Doxy). *α*-Tubulin levels are shown as a loading control. (**d** and **e**) Growth (**d**) and soft agar (**e**) assays of BE(2)-C_tetoff_MEIS2d in the presence or absence of Doxy. (**f**) Xenograft assay of BE(2)-C_tetoff_GFP and BE(2)-C_tetoff_MEIS2d cells. Tumor weight was analyzed by scatter plot with horizontal lines indicating the mean. Data (**b**, **d**, **e**, and **f**) were analyzed with two-tailed Student's *t*-test with the *P*-values indicated

Increased expression of mitotic genes is a common feature of cancers of advanced stages.^21^ Given that MEIS2 is essential for maintaining the expression of FOXM1, BMYB, and RBBP4, we examined the functional significance of increased MEIS2 expression in neuroblastoma cells. Transient overexpression of individual MEIS2 isoforms, with the exception of MEIS2a, increased mRNA expression of RBBP4, BMYB, and FOXM1, as well as their downstream target genes (Figure 6a). Importantly, FOXM1 depletion by shRNA significantly abolished the ability of MEIS2d to upregulate the expression of mitotic genes (Figure 6b), demonstrating that FOXM1 is an essential mediator of MEIS2 action in neuroblastoma cells. We chose MEIS2d for further functional studies, because it appeared to be the isoform most effective in transactivation of cell-cycle genes (Figure 6a). We established BE(2)-C cells with inducible expression of MEIS2d in the absence of doxycycline (Figure 6c). Induction of MEIS2d significantly enhanced neuroblastoma cell proliferation in culture (Figure 6d; Supplementary Figure S8), anchorage-independent growth in soft agar (Figure 6e), and tumorigenicity in immunodeficient mice (Figure 6f). These findings demonstrate an oncogenic activity of MEIS2 in neuroblastoma cells.

## Discussion

**Figure 7:**
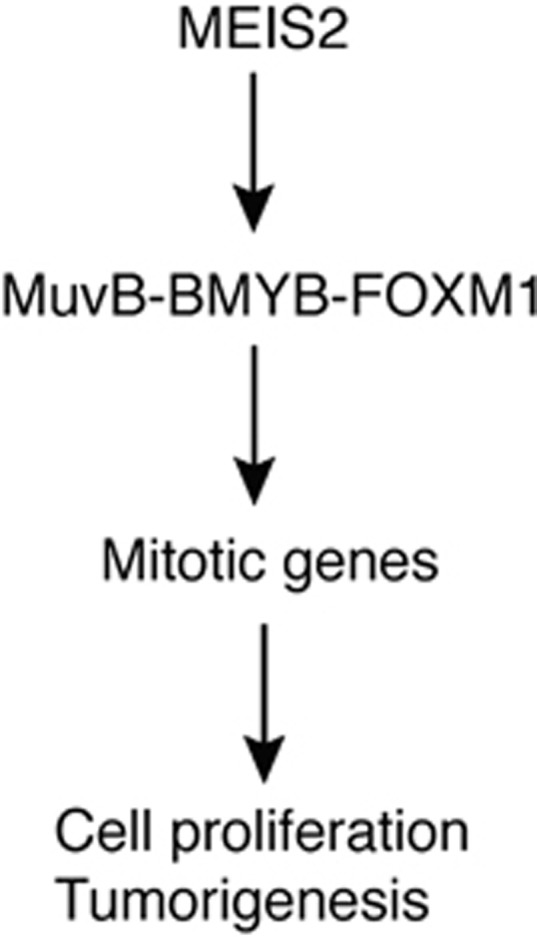
Model for MEIS2 transcriptional activation of the MuvB-RBBP4-FOXM1 complex to promote mitotic gene expression, cell proliferation, and tumorigenesis

In this report, we present evidence for an essential role of MEIS2, an important regulator of development, in the control of cell-cycle progression in neuroblastoma cells. We show that depletion of MEIS2 in neuroblastoma cells induces mitotic spindle aberrations, centrosome amplification, and M-phase arrest, leading to mitotic catastrophe, whereas ectopic MEIS2 expression enhances the proliferation and tumorigenicity of neuroblastoma cells. At the molecular level, MEIS2 depletion decreases the expression of late cell-cycle or mitotic genes, whereas MEIS2 overexpression increases their expression. We further provide evidence for MEIS2 as a key transcription activator of the MuvB-BMYB-FOXM1 complex, which functions as a master regulator of cell-cycle gene expression,^21^ thereby linking MEIS2 to the transcriptional control of cell-cycle genes and cell proliferation (Figure 7). Collectively, these findings suggest an oncogenic activity of MEIS2 in neuroblastoma pathogenesis and shed light on the molecular mechanism for the biological function of MEIS2 in development.

Cell-cycle progression is tightly linked to development that leads to the generation of a mature multicellular organism from the zygote. Accumulated evidence indicates a crucial role of cell-cycle genes in cell fate determination.^43^ However, the molecular mechanisms by which development genes regulate cell-cycle progression are less clear. It has been shown previously that RNAi-mediated knockdown of Meis2 in chick and mouse retinal progenitor cells inhibits cell proliferation, suggesting that Meis2 is essential for maintaining the progenitor cells in a rapidly proliferating state.^5^ Our results suggest a molecular mechanism for MEIS2 to promote cell proliferation during development by transcriptional activation of cell-cycle genes.

FOXM1 was originally identified as a transcription factor that activates the expression of many mitotic genes and is essential for execution of the mitotic program.^35, 36, 37^ High FOXM1 expression is a common feature of many types of cancers and is associated with poor prognosis.^20, 21^ FOXM1 is also overexpressed in primary neuroblastoma tumors and is essential for the tumorigenicity of neuroblastoma cells.^38^ Our study reveals that higher FOXM1 expression is significantly associated with advanced stages of neuroblastoma and decreased survival of neuroblastoma patients. The molecular mechanism underlying FOXM1 overexpression in various cancers is not well understood.^21^ We show that MEIS2 is essential for maintaining the expression of FOXM1 in neuroblastoma cells and *FOXM1* is a direct target gene of MEIS2, suggesting that high-level expression of MEIS2 is a major mechanism underlying FOXM1 overexpression in neuroblastoma.

The identification of *FOXM1* as a direct target gene of MEIS2 also offers a molecular explanation for the mitotic catastrophe phenotype induced by MEIS2 depletion. Mitosis is an ordered series of events, leading to equal distribution of duplicated chromosomes and centrosomes between the two daughter cells. Mitotic catastrophe can be triggered by aberrant mitosis resulting from defective G2-M checkpoint control, which promotes premature entry into mitosis,^31^ or from disruption of the mitotic machinery that ensures the correct segregation of chromosomes between daughter cells.^32^ FOXM1 target genes include those encoding protein components of both G2-M checkpoint (e.g., CDC25B and CHEK2) and the mitotic machinery for chromosome segregation (e.g., CENPA, CENPB, and CENPF). A majority of FOXM1 target genes, including CDC25B, CHEK2, CENPA, CENPB, and CENPF, were significantly downregulated in neuroblastoma cells with MEIS2 depletion. Their downregulation could disrupt the G2-M checkpoint control, the assembly of kinetochore, and the attachment of mitotic spindle microtubules to sister chromatids, thereby triggering mitotic catastrophe. Moreover, loss of FOXM1 also leads to centrosome amplification and mitotic catastrophe,^36^ a phenotype fully recapitulated by MEIS2 depletion. Together, these findings suggest that FOXM1 downregulation is a major mechanism for MEIS2 depletion-induced mitotic catastrophe. Nevertheless, overexpression of FOXM1 alone failed to rescue the cell death phenotype induced by MEIS2 depletion (data not shown). This is probably because MEIS2 is also required for the expression of BMYB and RBBP4, which form a complex with FOXM1 in transcriptional activation of mitotic genes.^21^

Finally, a key finding of our study is the identification of MEIS2 as an upstream transcription activator of BMYB and RBBP4 expression: MEIS2 depletion decreased their mRNA expression, whereas MEIS2 overexpression activated their transcription. We found no significant binding of MEIS2 to the promoters of *BMYB* and *RBBP4*, suggesting that MEIS2 activates their transcription indirectly. BMYB is a sequence-specific DNA-binding protein, and RBBP4 is a component of the MuvB complex that has an essential role in coordinating the expression of cell-cycle genes by sequentially interacting with and recruiting BMYB and FOXM1 to their target genes required for S- and M-phase progression, respectively.^21^ Dysregulation of the MuvB-BMYB-FOXM1 complex is frequently observed in cancers with poor prognosis and is thought to promote cancer progression by upregulating the expression of mitotic genes.^21^ The identification of MEIS2 as a transcription activator of the complex provides a molecular mechanism for the aberrant activation of mitotic genes in cancer cells and suggests a general oncogenic activity for MEIS2 in cancer development.

## Materials and Methods

### Cell lines and culture

The human neuroblastoma cell lines BE(2)-C (CRL-2268; ATCC, Manassas, VA, USA), SK-N-DZ (CRL-2149; ATCC), SK-N-F1 (CRL-2141; ATCC), and SHEP1^44^ cells were cultured in a 1 : 1 mixture of DMEM and Ham's nutrient mixture F12 supplemented with 10% fetal bovine serum (Invitrogen, Carlsbad, CA, USA). Cells were examined and phase-contrast images captured using an Axio Observer microscope and the software AxioVision (Carl Zeiss MicroImaging, Thornwood, NY, USA).

### Overexpression and RNA interference

The cDNA molecules coding for various human MEIS2 isoforms were generated by reverse transcription using total RNA prepared from the human neuroblastoma cell line BE(2)-C, verified by sequencing, and cloned into the lentiviral expression vector pCDH-CMV-MCS-puro (SBI, Mountain View, CA, USA). The Retro-X Tet-Off Advanced Inducible Gene Expression System (Clontech, Mountain View, CA, USA) was used to generate BE(2)-C cells with inducible MEIS2d expression in the absence of doxycycline. Myc-tagged human *MEIS2d* was generated by PCR using pCDH-puro-MEIS2d, verified by sequencing, and subcloned into pRetroX-Tight-pur. pLKO.1-based lentiviral constructs expressing shRNA to *MEIS2* (clone ID: TRCN0000016043, TRCN0000016044, TRCN0000016045, TRCN0000016046, and TRCN0000016047) were purchased from Open Biosystems (Huntsville, AL, USA) and to *FOXM1* (clone ID: TRCN0000273984, TRCN0000015543, TRCN0000015544, TRCN0000015545, and TRCN0000015546) from Sigma-Aldrich (St. Louis, MO, USA). pLKO.1-shGFP (Addgene, Cambridge, MA, USA) was used as a control. Retroviruses and lentiviruses were produced in 293FT cells using the packaging plasmids pHDM-G and pMD.MLVogp (retroviruses) or pLP1, pLP2, and pLP/VSVG (lentiviruses).

### Cell-cycle and survival assays

For cell-cycle analysis, cells were fixed in 70% ethanol, incubated with ribonuclease A (Sigma-Aldrich), and stained with 20 *μ*g/ml propidium iodide (Invitrogen). Samples were analyzed using a FACSCalibur system and ModFitLT V3.2.1 software (BD Bioscience, San Jose, CA, USA). Cell viability was measured by trypan blue exclusion assay. Genomic DNA fragmentation assay was performed as previously described.^45^

### Immunoblotting and Immunofluorescence

Immunoblotting was conducted according to standard procedures using the following primary antibodies: rabbit anti-BCL2 (sc-783, 1 : 200), rabbit anti-caspase 3 (sc-7148, 1 : 200), rabbit anti-Flag (F7425, 1 : 1000; Sigma-Aldrich), rabbit anti-FOXM1 (sc-502, 1 : 100), rabbit anti-GAPDH (sc-25778, 1 : 3000), mouse anti-MEIS2 (sc-81986, 1 : 400), rabbit anti-*β*-actin (600-401-886, 1 : 2000; Rockland Immunochemicals, Gilbertsville, PA, USA), and mouse anti-*α*-tubulin (B-5-1-2, 1 : 5000; Sigma-Aldrich). Unless indicated, all primary antibodies were purchased from Santa Cruz Biotechnology (Dallas, TX, USA). Horseradish peroxidase-conjugated goat anti-mouse and goat anti-rabbit IgG (Santa Cruz Biotechnology) were used as secondary antibodies. Proteins were visualized using a SuperSignal West Pico chemiluminescence kit (Pierce, Rockford, IL, USA) and quantified using ImageJ (version 1.47d, NIH, Bethesda, MD, USA). Films were exposed for various times for quantification of target proteins within their linear range of detection. For visualization and quantification with the Odyssey system, goat anti-mouse IRDye 800, anti-rabbit IRDye 800, anti-mouse IRDye 680, and anti-rabbit IRDye 680 were used as secondary antibodies (LI-COR Biosciences, Lincoln, NE, USA). For immunofluorescence, cells were fixed for 10 min either with 4% paraformaldehyde at room temperature or with a 1 : 1 mixture of acetone and methanol for at −20 °C (for *γ*-tubulin), and stained with mouse anti-*α*-tubulin (B-5-1-2, 1 : 2000), mouse anti-*γ*-tubulin (GTU-88, 1 : 1000; Sigma-Aldrich), and rabbit anti-phospho-histone H3 (06-570, 1 : 200; Millipore, Billerica, MA, USA). All secondary fluorescence antibodies were from Molecular Probes (Grand Island, NY, USA) and used at 1 : 800 dilutions for goat anti-mouse (Alexa Fluor 488) and goat anti-rabbit (Alexa Fluor 594). Nuclei were stained with DAPI. Fluorescent images were captured with a fluorescence microscope and the software AxioVision (Carl Zeiss Axio Observer, Carl Zeiss Microscopy, Thornwood, NY, USA).

### Microarray gene expression profiling

Total RNA was isolated using Trizol (Invitrogen) from three replicate samples of BE(2)-C cells infected with lentiviruses expressing either shGFP or shMEIS2-43 for 48 h. Affymetrix microarray was performed using the Human Gene 1.0 ST microarray chip (Agilent Technologies, Santa Clara, CA, USA). Data were normalized, significance determined by ANOVA, and fold change calculated with the Partek Genomics Suite (Partek Inc., St. Louis, MO, USA). GO analysis was performed with DAVID^46^ for all differentially expressed genes (≥+1.5 and ≤−1.5 fold, *P*<0.01). Microarray data were also analyzed by gene set enrichment analysis (GSEA).^47^ The microarray data have been deposited in the NCBI Gene Expression Omnibus (GEO) with the accession number GSE56003 (http://www.ncbi.nlm.nih.gov/geo/query/acc.cgi?acc=GSE56003).

### Quantitative RT-PCR

Total RNA was isolated from three replicate samples using Trizol. Reverse transcription was performed using SuperScript II Reverse Transcriptase (Invitrogen). Quantitative real-time PCR was performed using a RT^2^ SYBR green/Fluorescein PCR master mix (SABiosciences, QIAGEN, Valencia, CA, USA) on an iQ5 real-time PCR system (Bio-Rad, Hercules, CA, USA) with primers against *BMYB* (*MYBL2*), *CCNB1, CDC25A, CDC25B, CDCA3, CDK2, CENPA, CHEK1, CHEK2, FOXM1, MEIS2, RBBP4, SPC24,* and *B2M* (*β*2 microglobulin) (Supplementary Table S1). All primer pairs were verified by melting curve analysis following qRT-PCR, with each primer pair showing a single desired amplification peak. All samples were normalized to B2M mRNA levels.

### Chromatin immunoprecipitation-qPCR

Each ChIP sample used 4 × 10^7^ BE(2)-C cells. Cross-linked chromatin DNA was sheared through sonication and immunoprecipitated using mouse anti-MEIS2 (sc-81986) or normal mouse IgG (sc-2025), all from Santa Cruz Biotechnology, according to the published procedure.^48^ ChIP genomic DNA samples were assayed in triplicate by PCR using an iQ5 real-time PCR system (Bio-Rad) and primer sets that cover the following *FOXM1* promoter regions: FOXM1p-779 (−540 to −779), TGCTGGGATTACAGGCGTGAG, CTCCCGAAGCCAAGCCTTCGG; FOXM1p-312 (−158 to −312), GTGTCGCCTGGCGTGACCAGC, AAGAAGTGGCCGTGGGGCCGG.

### Soft agar and xenograft assays

Cells (1000/well) were mixed with 0.3% Noble agar in DMEM growth medium and plated onto six-well plates containing a solidified bottom layer (0.6% Noble agar in DMEM growth medium). After 14 days, colonies were stained with 5 mg/ml MTT (Sigma-Aldrich), photographed and counted. For xenograft assay, 6-week-old female NOD.SCID/NCr mice (NCI, Frederick, MD, USA) were injected subcutaneously at both flanks with 5 × 10^6^ BE(2)-C_tetoff or BE(2)-C_tetoff_MEIS2d cells in 200 *μ*l serum-free DMEM. Eighteen days after injection, tumors were removed and weighed. All animal experiments were pre-approved by the Institutional Animal Care and Use Committee of Georgia Regents University.

### Analyses of patient data

Patient data used in this study were described previously.^49^ Gene expression data sets were obtained from the Oncogenomics Database (http://pob.abcc.ncifcrf.gov/cgi-bin/JK).^50^ Kaplan–Meier analysis was conducted online, and the resulting survival curves and *P*-values (log-rank test) were downloaded. All cutoff values for separating high and low expression groups were determined by the online Oncogenomics algorithm.^50^

### Statistical analysis

For immunofluorescence staining, ∼400–1000 cells (DAPI positive) were counted from at least 10 random × 630 fields, and the percentages of phospho-histone H3^+^ cells without or with multipolar spindles or centrosomes were determined. Statistics were determined with unpaired, two-tailed Student's *t*-test using Prism 6.01 for Mac (GraphPad Software, La Jolla, CA, USA), and a *P–*value of <0.05 is considered to be statistically significant.

## Abbreviations

ChIP: chromatin immunoprecipitation
FDR: false discovery rate
FOXM1: Forkhead box protein M1
GSEA: gene set enrichment analysis
GO: gene ontology
Hth: homothorax
MEIS2: Myeloid ecotropic insertion site 2
MuvB: multi-vulval class B
MYBL2/BMYB: v-myb avian myeloblastosis viral oncogene homolog-like 2
qPCR: quantitative reverse transcription PCR
RBBP4: retinoblastoma binding protein 4
shRNA: small-hairpin RNA
TALE: three amino-acid loop extension
TGF-*β*: transforming growth factor *β*
