# Supplementary material for: MEIS2 is essential for neuroblastoma cell survival and proliferation by transcriptional control of M-phase progression
Source: Cell Death Dis. 2014 Sep 11;5(9):e1417–. doi: 10.1038/cddis.2014.370 (PMC4540202; doi:10.1038/cddis.2014.370)
Supplement: Supplementary Information [file cddis2014370x1.pdf]

MEIS2 is essential for neuroblastoma cell survival and proliferation by transcriptional control of M phase progression

Yunhong Zha[1], Yingfeng Xia[1,2], Jane Ding[2], Jeong-Hyeon Choi[2,3], Liqun Yang[7], Zheng Dong[4], Chunhong Yan[2,5], Shuang Huang[5], and Han-Fei Ding[2,6]

[1]Department of Neurology, the First Hospital of Yichang, Three Gorges University College of Medicine, Yichang, 443000, China; [2]Cancer Center, [3]Department of Biostatistics and Epidemiology, [4]Department of Cellular Biology and Anatomy, [5]Department of Biochemistry and Molecular Biology, [6]Department of Pathology, Medical College of Georgia, Georgia Regents University, Augusta, Georgia 30912, USA; [7]State Key Laboratory of Silkworm Genome Biology, Institute of Sericulture and System Biology, Southwest University, Chongqing, 400716, China

Supplementary Figures S1-S8
Supplementary Table S1

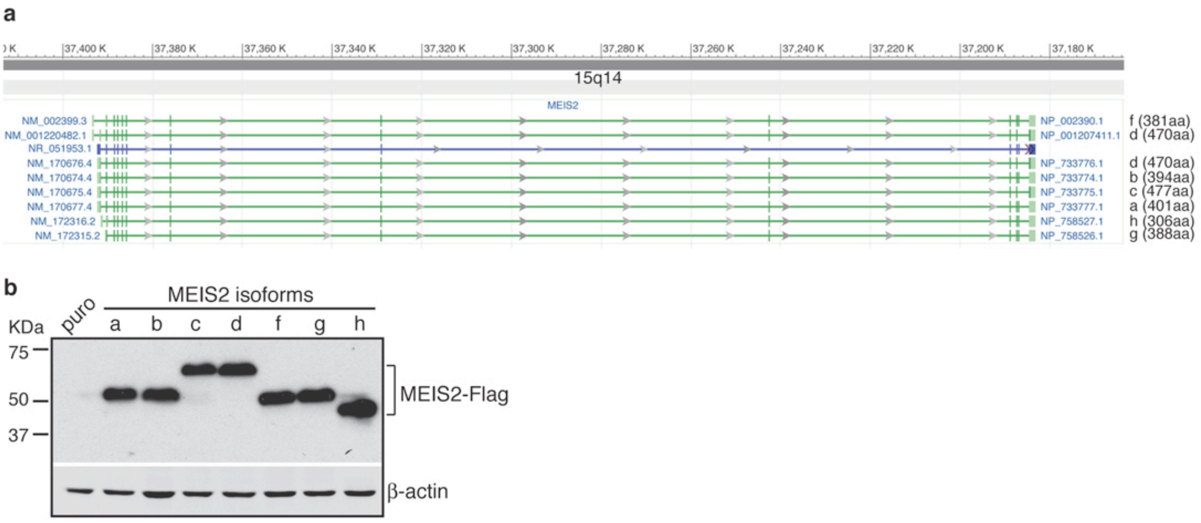

Figure S1. MEIS2 isoforms. (**a**) The human *MEIS2* gene and its isoforms (http://www.ncbi.nlm.nih.gov/gene/4212). The number of amino acid residues for each isoform is indicated. (**b**) Immunoblotting of Flag-tagged MEIS2 isoforms in 293FT cells transfected with pCDH-puro-based lentiviral constructs expressing individual MEIS2 isoforms. β-actin levels are shown as loading control.

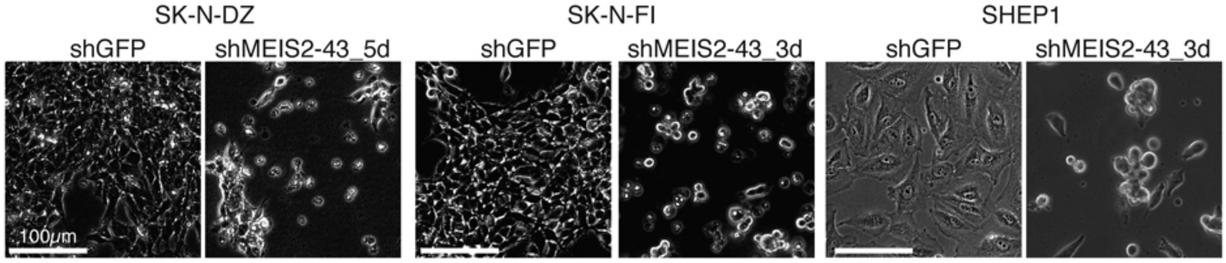

Figure S2. MEIS2 depletion induces cell death in the human neuroblastoma cell lines SK-N-DZ, SK-N-FI, and SHEP1.

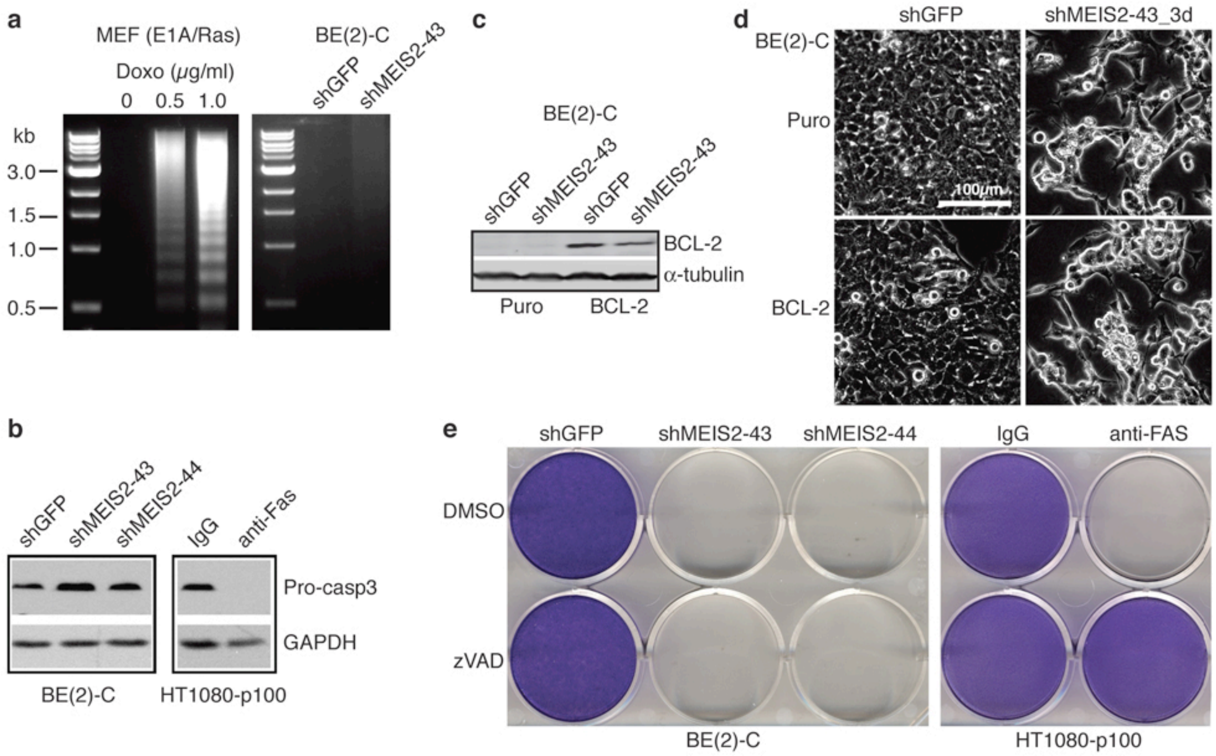

Figure S3. MEIS2 depletion induces non-apoptotic cell death. (**a**) DNA fragmentation assay showing no apoptosis-induced genomic DNA fragments in BE(2)-C cells following MEIS2 depletion. E1A/Ras-transformed mouse embryo fibroblasts (MEF), which are highly sensitive to the DNA-damage drug doxorubicin (Doxo), were used as positive control for apoptosis-induced genomic DNA fragmentation. (**b**) Immunoblotting showing no cleavage of pro-caspase 3 in BE(2)-C cells following MEIS2 depletion. The human fibrosarcoma HT1080 cells expressing NF-κB2 p100, which are highly sensitive to anti-Fas (CH11, 100 ng/ml)-triggered apoptosis, were used as positive control. (**c**) Immunoblotting of BCL-2 in BE(2)-C cells with or without BCL-2 overexpression or MEIS2 depletion. (**d**) Phase-contrast images showing no protection of MEIS2 depletion-induced cell death by BCL-2. (**e**) Crystal violet staining showing no protection of MEIS2 depletion-induced cell death by zVAD-FMK (10 μm). Anti-Fas-induced apoptosis was used as positive control.

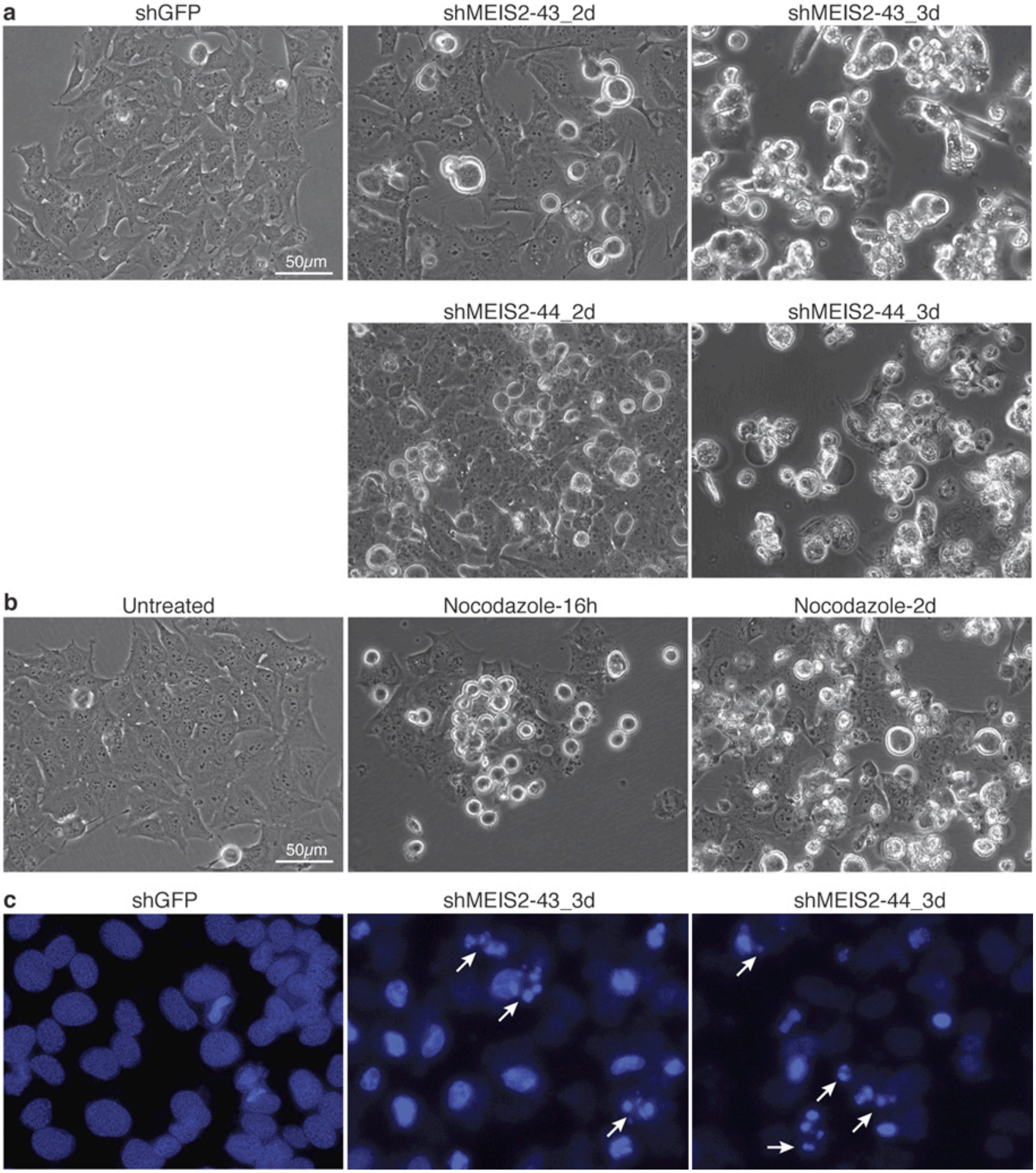

Figure S4. MEIS2 depletion induces cell death with micronucleation. (**a-b**) Phase-contrast images of BE(2)-C cells with MEIS2 depletion (**a**) or treated with Nocodazole (**b**), an anti-cancer agent that induces M phase arrest by interfering with the polymerization of microtubules. (**c**) Hoechst 33342 staining of nuclei showing chromatin condensation and micronucleation (arrows) in BE(2)-C cells following infection with lentiviruses expressing shMEIS2-43 or shMEIS2-44.

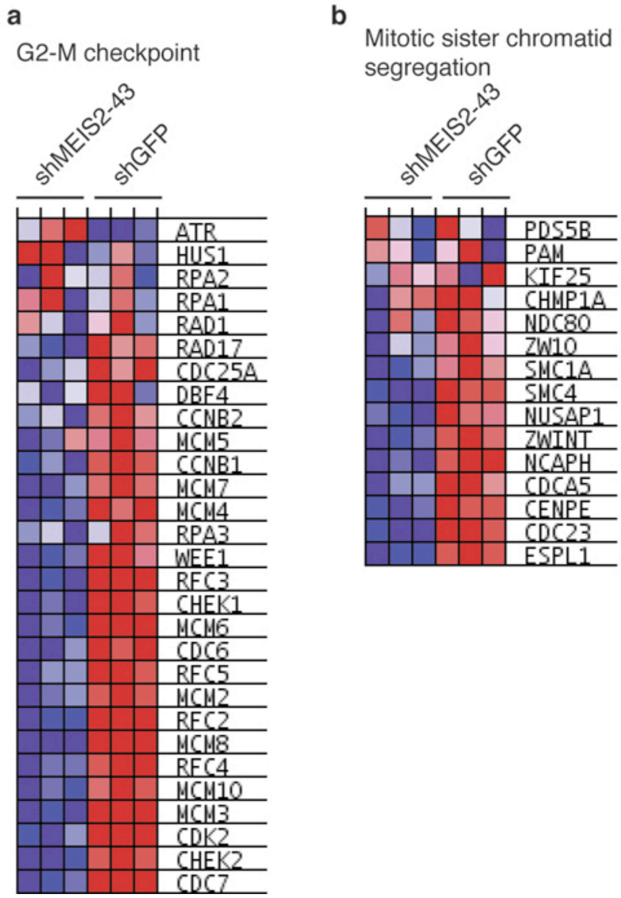

Figure S5. GSEA showing downregulation of genes involved in the G2-M checkpoint control (**a**) and mitotic sister chromatid segregation (**b**).

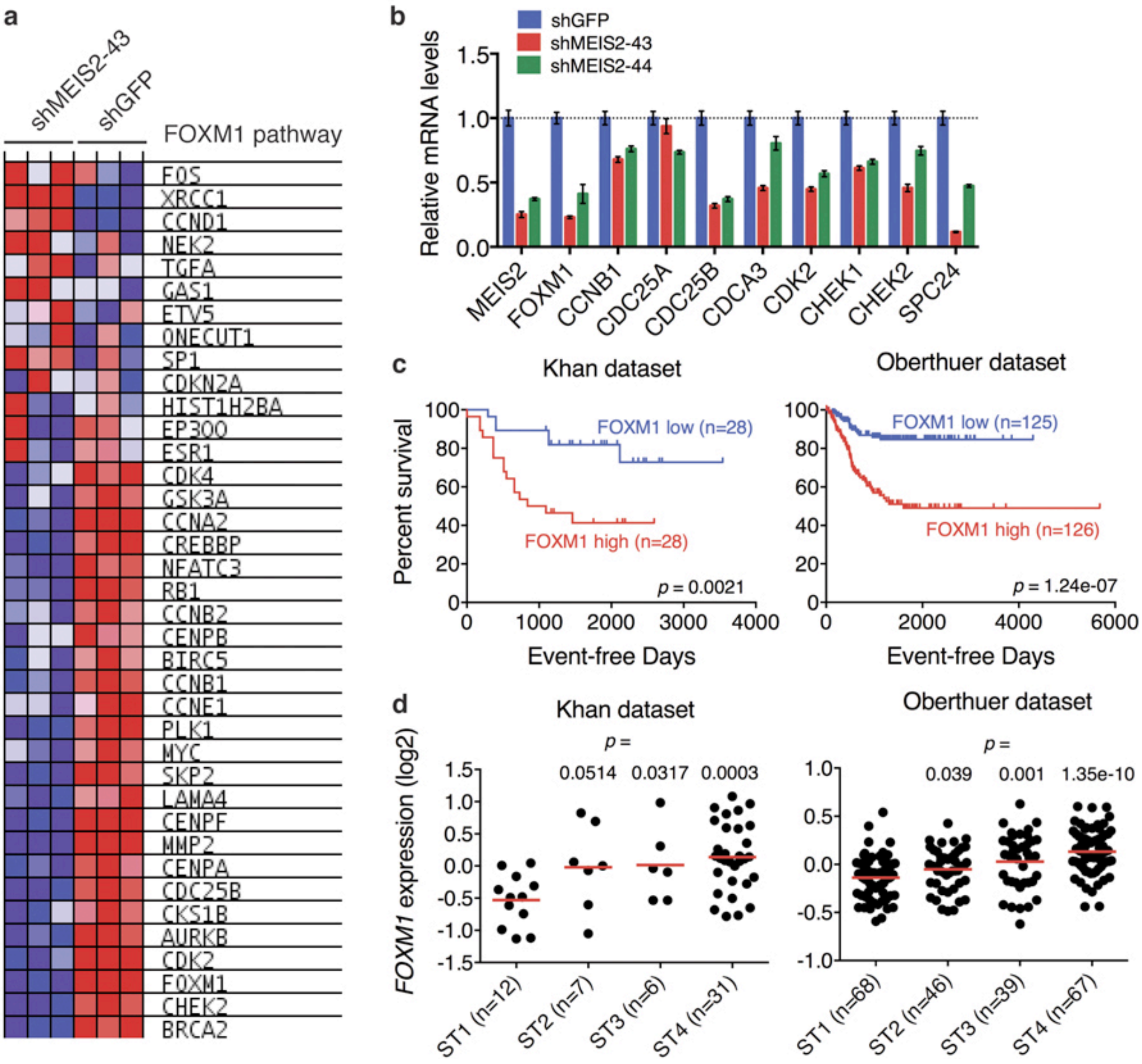

Figure S6. MEIS2 is essential for maintaining FOXM1 expression in neuroblastoma cells. (**a-b**) GSEA (**a**) and qRT-PCR analysis (**b**) showing downregulation of FOXM1 and its downstream target genes in BE(2)-C cells with MEIS2 depletion. Error bars, s.d. (n = 3). (**c-d**) High FOXM1 expression is significantly associated with poor prognosis (**c**) and advanced tumor stages (**d**) in neuroblastoma patients. Data in (**c**) were analyzed with the log-rank test with *p* values indicated, and data in (**d**) (ST1 vs ST2, 3, or 4) were analyzed with two-tailed Student's *t*-test with *p* values indicated.

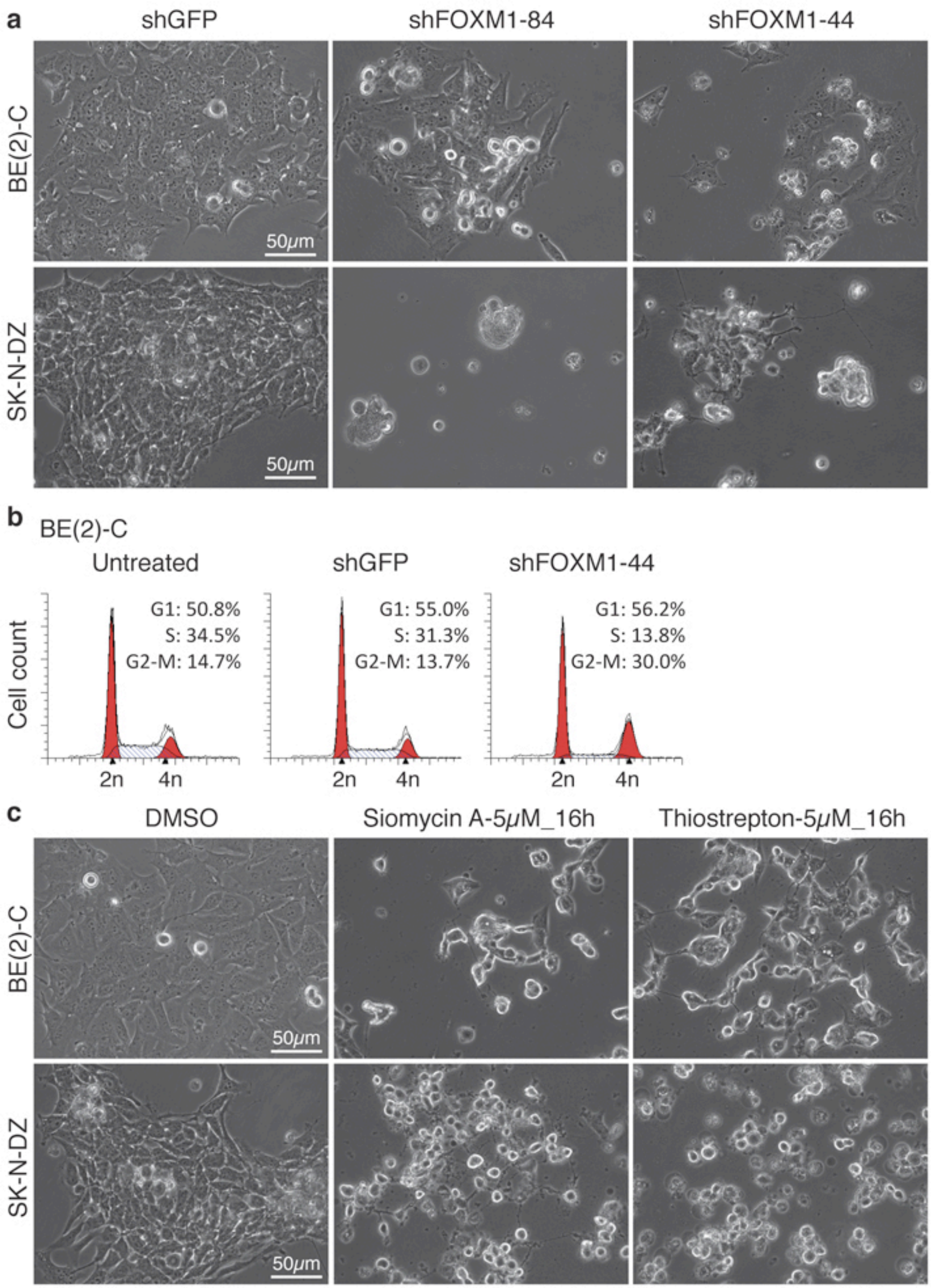

Figure S7. FOXM1 depletion or inhibition induces cell death with M phase arrest in neuroblastoma cells. (**a**-**b**) Micrographs (**a**) and cell cycle analysis of indicated neuroblastoma cell lines with FOXM1 depletion. (**c**) Micrographs of indicated neuroblastoma cell lines treated with FOXM1 inhibitors.

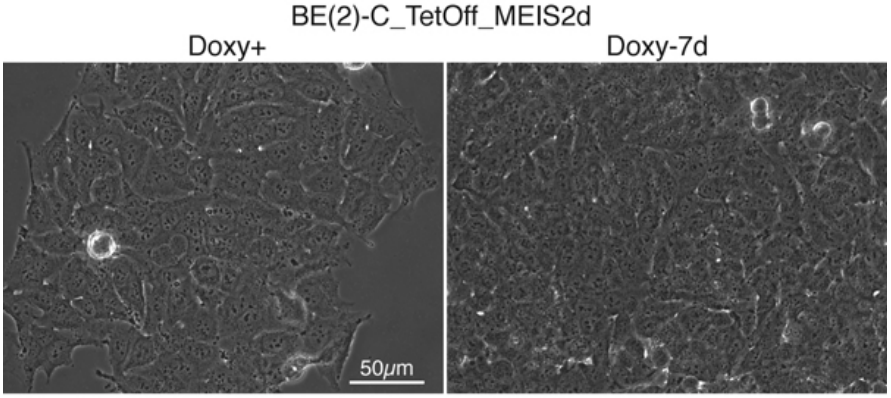

Figure S8. Micrographs of neuroblastoma BE(2)-C cells with or without iducible MEIS2d overexpression.

Table S1. RT-PCR primers

Primer set	Forward	Reverse
BMYB	CACCAGAAACGAGCCTGCCTTA	CTCAGGTCACACCAAGCATCAG
CCNB1	GACCTGTGTCAGGCTTTCTCTG	GGTATTTTGGTCTGACTGCTTGC
CDC25A	TCTGGACAGCTCCTCTCGTCAT	ACTTCCAGGTGGAGACTCCTCT
CDC25B	AGAACCTCCTGGACAGTGACCA	GCTGAACTTGCCCGTCAATAGG
CDCA3	GTTCACCTAGTGCTGGCATCCT	GGATCTGAGTCCTGGGCATGTT
CDK2	ATGGATGCCTCTGCTCTCACTG	CCCGATGAGAATGGCAGAAAGC
CENPA	GGCGGAGACAAGGTTGGCTAAA	GGCTTGCCAATTGAAGTCCACAC
CHEK1	GTGTCAGAGTCTCCCAGTGGAT	GTTCTGGCTGAGAACTGGAGTAC
CHEK2	GACCAAGAACCTGAGGAGCCTA	GGATCAGATGACAGCAGGAGTTC
FOXM1	ACTTTAAGCACATTGCCAAGC	CGTGCAGGGAAAGGTTGT
MEIS2	GACCACGATGATGCAACC	CCTGTGTCTTGCGCTAACTG
RBBP4	AGACTTGCGTCTCCGTGGACAT	CCTCCTTTGGAACGGCACTGAT
SPC24	GGGATTATGAGTGTGAGCCAGG	ACTCCAGAGGTAGTCGCTGATG
B2M	TGCTGTCTCCATGTTTGATGTATCT	TCTCTGCTCCCCACCTCTAAGT
